# Design of Azomethine Diols for Efficient Self-Healing of Strong Polyurethane Elastomers

**DOI:** 10.3390/molecules23112928

**Published:** 2018-11-09

**Authors:** Dae-Woo Lee, Han-Na Kim, Dai-Soo Lee

**Affiliations:** Division of Semiconductor and Chemical Engineering, Chonbuk National University, Baekjedaero 567, Jeonju 54896, Korea; dwlee2310@hanmail.net (D.-W.L.); hnk07@hanmail.net (H.-N.K.)

**Keywords:** azomethine diol, azoemtheine metathesis, self-healing efficiency, mechanical property

## Abstract

Azomethine diols (AMDs) were synthesized by condensation between a terephthalic aldehyde, polyether diamine, and ethanol amine. The synthesized AMDs were employed to introduce azomethine groups into the backbones of polyurethane elastomers (PUEs). Different AMDs were designed to control the concentration and distribution of azomethine groups in PUEs. In this study, we explored the intrinsic self-healing of AMD-based PUEs by azomethine metathesis. Particularly, the effects of the concentration and distribution of the azomethine groups on the AMD-based PUEs were considered. Consequently, as the azomethine group concentration increased and the distribution became denser, the self-healing properties improved. With AMD3-40, the self-healing efficiency reached 86% at 130 °C after 30 min. This represents a 150% improvement over the control PUE. Additionally, as the AMD content increased, the mechanical properties improved. With AMD3-40, the tensile strength reached 50 MPa. Therefore, we concluded that the self-healing and mechanical properties of PUEs can potentially be tailored for applications by adjusting the concentration and design of AMD structure for PUEs.

## 1. Introduction

Polyurethane elastomer (PUE) is one of the most versatile industrial polymers. PUEs are synthesized via a polyaddition reaction between polyols, diisocyanates, and chain extenders. PUEs with various mechanical properties can be synthesized by combining different polyols and diisocyanates. The soft domain of PUE consists of polyol, while its hard domain comprises isocyanate and chain extender. The polyol in the soft domain endows elastic properties, and the strong hydrogen bonds between the hard domains confer rigidity to the PUE. Thus, the soft domain and hard domain contents significantly affect the mechanical properties of PUE. Several researchers reported chain scission of PU at an upper stability temperature of about 200 °C [1,2,3]. PU has traditionally been widely used for foams for thermal insulation and shock-absorbance, coatings, adhesives, fibers, and elastomers [4,5,6,7].

However, recently, studies have been conducted on the shape memory and self-healing properties of PUEs. Many researchers studied self-healing polymers to improve the durability and applicability of polymer products. Several methods have been suggested for self-healing polymers. In the early stage of self-healing polymers, self-healing methods based on microcapsules have been extensively studied as an extrinsic method [8]. During crack propagation, the microcapsules are broken, releasing a healing agent. However, a disadvantage of self-healing by an extrinsic method is the reagent stored in the microcapsule is unstable, making repetitive self-healing impossible. For repetitive self-healing, the healing agent should be maintained in an uncured state. However, since the healing agent is cured after the first healing, repetitive self-healing is impossible.

To address these problems, intrinsic self-healing methods are proposed. These can be classified into two types. The first is associative and occurs via hydrogen bonding, host–guest interactions, or exchange reactions involving disulfide metathesis, transesterification, transcarbamoylation, transalkylation or azomethine metathesis [9,10,11,12,13]. The second is dissociative, involving reversible Diels–Alder reactions and urethane or urea group reactions [14,15,16,17,18]. However, generally, self-healing and mechanical properties show an inverse relationship. Self-healing polymers employing aromatic disulfide groups can self-heal even at room temperatures but have poor mechanical properties. In this category, hydrogels and supramolecular polymers have been studied extensively [18,19,20,21]. Self-healing polymers employing reversible urethane groups undergo self-healing only above 200 °C, which is the dissociation temperature of reversible urethane groups. In the case of polymers using hindered urea groups, the bulky structure around the urea group may lower the dissociation temperature and allow self-healing at room temperature. However, self-healing polymers employing reversible urethane or urea groups show relatively poor mechanical properties [17,18]. Self-healing is affected by various factors that can also influence the flow properties of polymers, hydrogen bonding, phase mixing, and crystallization. In self-healing using disulfide, the toughness is below 15 MJ/m [22]. Recently, polymers that exhibit self-healing properties at low temperatures while maintaining excellent physical properties are reported. The time required for a full scratch recovery of a polyurethane film that comprises poly(tetramethylene ether glycol), isophorone diisocyanate, and aromatic disulfide is only 30 min at 40 °C [22]. According to Yanagisawa and co-workers, the self-healing of urethane is completed at 36 °C after 24 h via the hydrogen bonding of thiourea [23].

An azomethine has the general structure (–C=N–) and can undergo rapid exchange with no side reactions [24,25,26,27]. Generally, the azomethine group is formed by condensation between amine and aldehyde. Kaya and co-workers studied its optical and electrical applications by introducing azomethine into a urethane backbone; however, they did not consider self-healing of azomethine-based PUs [28,29,30]. Jie and co-worker synthesized polyazomethine and confirmed its self-healing properties via the exchange reaction of imine groups, imine metathesis [31]. Many researchers reported malleable polyazomethine. Studies on metal complexes with azomethine groups with respect to their membrane applications are also conducted [9,12]. Leibler and co-workers reported that the self-healing properties of polymers are closely related to their relaxation time [32,33,34]. In this study, azomethine groups were introduced into the main chain of PUE to study self-healing via exchange reactions of azomethine metathesis. Particularly, the effects of the concentration and distribution of the azomethine structure on self-healing were investigated. By adjusting the concentration and distribution of the azomethine structure, the self-healing properties can potentially be tailored to applications. Thus, it is believed that the azomethine diol (AMD) based PUE used in this study can be employed in self-healing high performance PUEs. In particular, it is expected that it can be applied to improve the processability of thermoplastic elastomers and hot-melt adhesives.

## 2. Results and Discussion

### 2.1. Preparation of AMD-Based PUEs

**Scheme 1:**
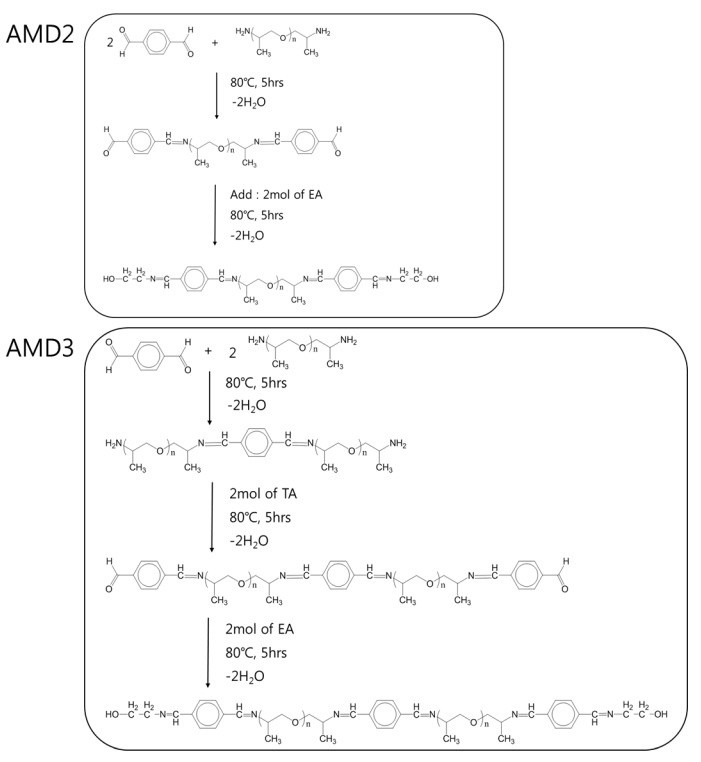
Synthetic processes and structures of AMD2 and AMD3.

**Figure 1:**
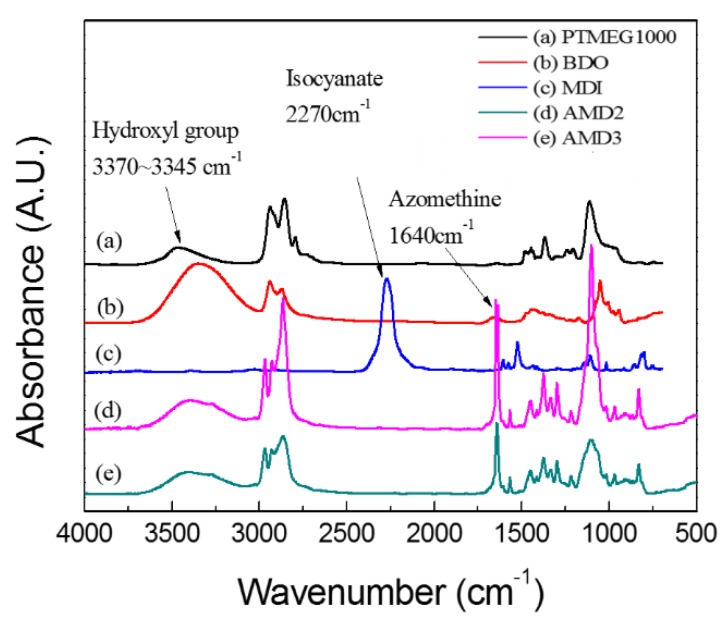
FT-IR spectra of raw materials and synthesized AMDs.

AMDs, AMD2 and AMD3, synthesized via different processes from terephthalaldehyde (TA), polyether diamine, and ethanol amine (EA) are illustrated in Scheme 1. There are six azomethine groups in one AMD3 while there are four azomethine groups in one AMD2. It is postulated that the azomethine groups in AMD3 based PUEs are denser than those in AMD2 based PUEs at the same concentrations of azomethine groups in PUEs. Figure 1 shows the FT-IR spectra of the raw materials, poly (tetramethylene ether) glycol (PTMEG, MW 1000) (PTMEG1000), and 1,4-butanediol (BDO) and 4,4′-methylene diphenyl diisocyanate (MDI) as well as AMDs, used for the synthesis of PUEs. Characteristic absorptions due to the stretching vibration of azomethine in AMDs were observed at 1640 cm^−1^. Absorptions around 3360 and 2933 cm^−1^ were attributed to the –OH and –CH vibrations of AMDs, respectively. Additionally, the hydroxyl values of AMD2 and AMD3 were 204.1 and 126.8 mg KOH/g, respectively. The hydroxyl value was determined using the ASTM 4274D method. Figures S1 and S2 display the ^1^H-NMR spectra of AMD2 and AMD3, respectively. Protone peaks of azomethine and benzene ring were observed at 8.3 and 7.8 ppm, respectively.

**Figure 2:**
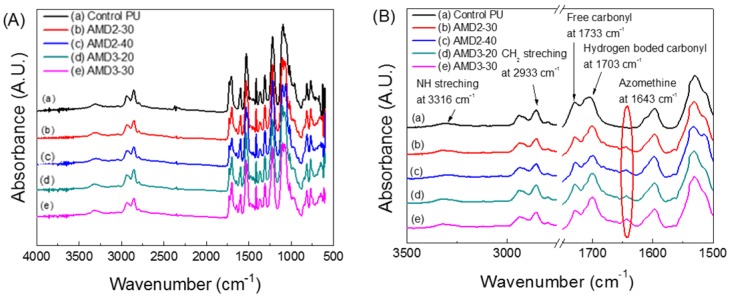
FT-IR spectra of prepared AMD based PUEs: (**A**) full spectra; and (**B**) magnified spectra.

PUEs were prepared from prepolymers following the processes shown in Figure S3 and the sample codes of the synthesized PUEs are given in Table 1. After curing the PUEs at 110 °C for 24 h, complete disappearance of peaks at 2270 cm^−1^ due to the reaction could be confirmed by FT-IR spectroscopy, as shown in Figure 2. Figure 2A shows FT-IR spectra of representative PUEs prepared with AMDs. In magnified spectra shown of Figure 2B, weak absorption peaks attributed to azomethine groups appear at 1643 cm^−1^ for the PUEs. The PUEs prepared with AMDs showed stronger absorptions of the hydrogen-bonded carbonyl groups at 1703 cm^−1^ compared with the control PU. The –NH– stretching and –CH absorptions of PUs were comparable. Table 2 summarizes the average molecular weights of PUEs determined by gel permeation chromatography (GPC). The average molecular weights of the PUs synthesized in this study were similar.

### 2.2. Thermal Analyses of PUEs Based on AMD

**Figure 3:**
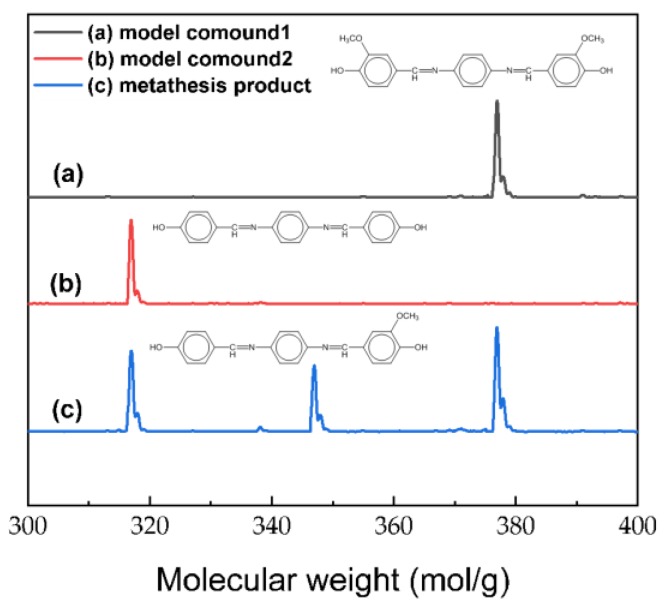
LC-MS results for the metathesis of azomethine diols of different structures: (**a**) Model Compound 1; (**b**) Model Compound 2; and (**c**) metathesis products of model compounds.

Thermogravimetric analysis (TGA) was used to investigate the AMD2- and AMD3-based PUEs (Figure S4). Thermal stability of synthesized PUEs decreased with increase in azomethine content. However, weight loss due to the formation of volatile small molecules by thermal degradation was not observed below 200 °C. The results from TGA are included in Table S3. According to Zhang, imine metathesis at elevated temperature induced self-healing and malleability in crosslinked polyimine [35,36]. LC-MS is a useful analytical method to confirm an exchange reaction of metathesis [37,38,39]. The azomethine metathesis of AMDs was studied with mixtures of model compounds of AMDs prepared from 1,4-phenylene diamine and hydroxybenzyl aldehydes of different structures. Figure 3 shows LC-MS data of two model compounds, and mixtures after the metathesis. After stirring the mixtures at 60 °C for 5 h, they showed the characteristic peaks of the metatheses products.

**Figure 4:**
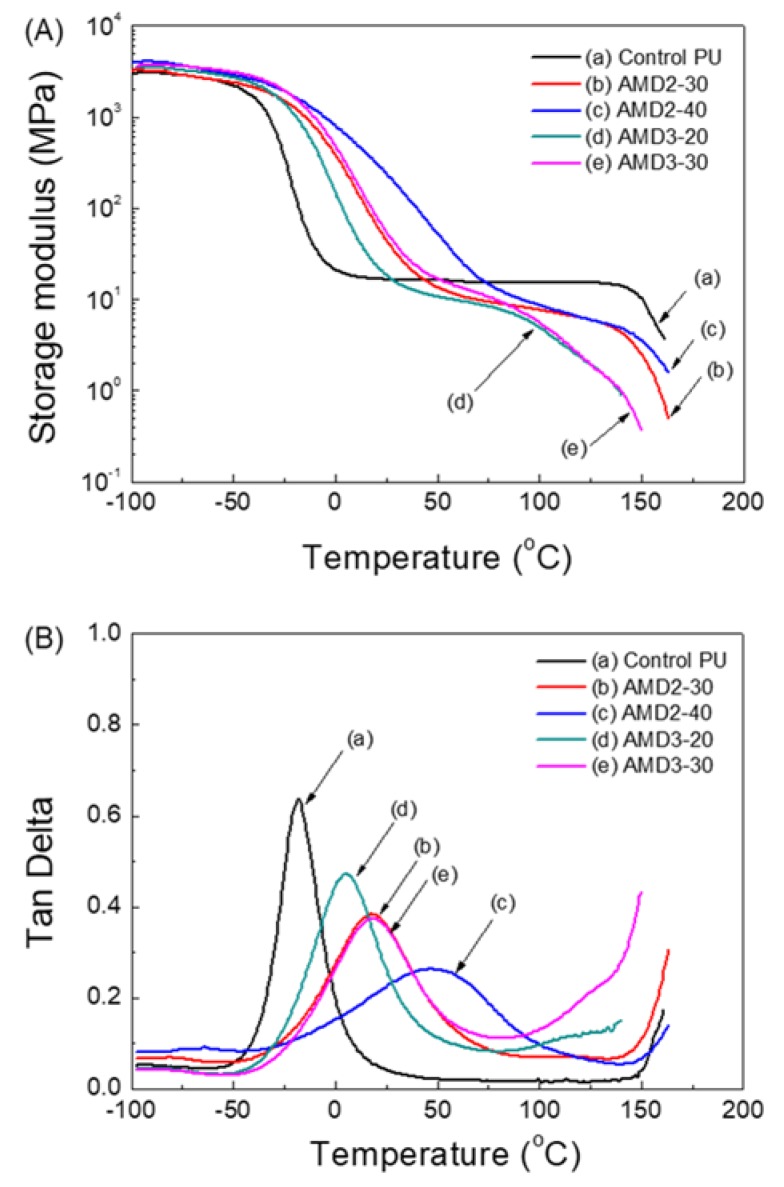
DMA thermograms of AMD-based PUs: (**A**) storage moduli of prepared AMD-based PUs; and (**B**) tan delta of prepared AMD-based PUs.

Dynamic mechanical analysis was performed to study the thermal properties of PUEs and thermograms are displayed in Figure 4 and Figure S5. The soft segment domains of the PUEs underwent glass–rubber transitions below room temperature. The peak temperatures of tan delta curves were considered as glass transition temperatures of soft segment domains (T_gs_) in PUEs. The T_gs_ increased as the concentration of AMDs in PUEs increased. The increase of T_gs_ implies the incorporation of AMD segments into the soft segment domains. The flow temperature (T_flow_), at which the storage moduli of the PUEs decreased after the rubbery plateau region, was lowered as the AMD content in the PUEs increased due to the metathesis of azomethine groups in soft domains. It is worth noting that AMD2-30 and AMD3-20, and AMD2-40 and AMD3-30, contained similar concentrations of azomethine groups in Table 1, while the azomethine groups in AMD3-20 and AMD3-30 are closer than in AMD2-30 and AMD2-40. Thus, azomethine metathesis of AMD3-20 and AMD3-30 are expected to be more efficient than those of AMD2-30 and AMD2-40 and the T_flow_ of AMD3-20 and AMD3-30 are lower than those of AMD2-30 and AMD2-40. Differential scanning calorimetry (DSC) was used to investigate the AMD2- and AMD3-based PUEs. (The results of DSC are displayed in Figure S6 and summarized in Table S3.) In DSC thermograms, T_gs’_s below room temperature increased with increase in AMD content and the melting temperatures of the hard domains near 140 °C changed little. This result is due to the phase mixing between the soft segment domains and AMD segments. Small-angle X-ray scattering (SAXS) patterns are given in Figure S7. The interdomain distance decreased from 20.8 to 18 nm as the content of AMD increased and shoulders appeared due to further decrease of the interdomain distance to 12 nm partially. Lai et al. also reported that, in AMD-based PUs, interdomain distance was decreased to 12.8 nm [40]. The AFM images in Figure S8 shows a similar trend, i.e., a decrease in the hard domain size with increasing AMD content, resulting in the decrease of interdomain distances. These results imply a decrease in micro-phase separation with increasing AMD content in PUEs.

**Figure 5:**
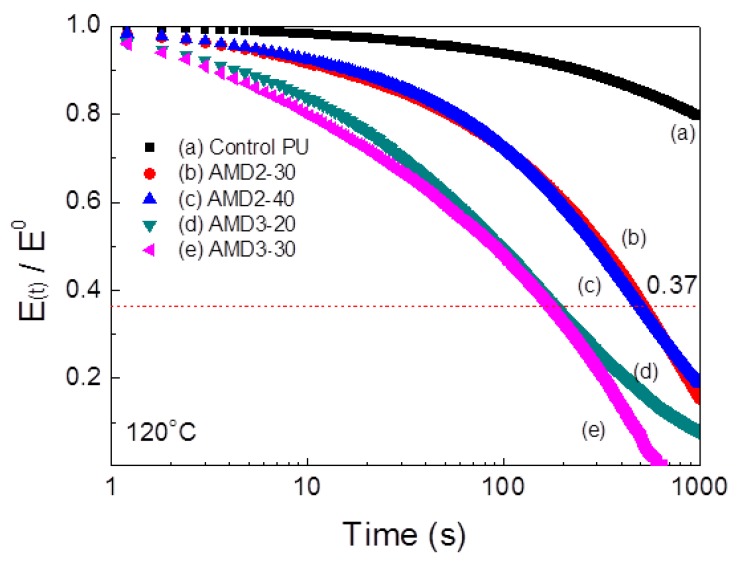
Typical normalized stress relaxation behaviors of AMD-based PUEs.

**Figure 6:**
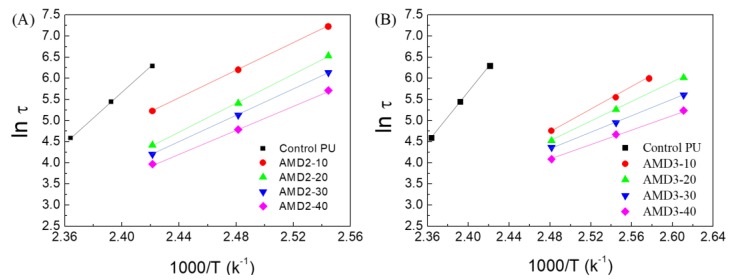
Arrhenius plots for relaxation times of AMD-based PUEs: (**A**) AMD2 based PUEs; and (**B**) AMD3 based PUEs.

The self-healing of polymers was investigated frequently employing the stress relaxation tests [20,21,22,23]. The stress relaxation of PUEs was investigated using dynamic mechanical analyzer (DMA) and results of the stress relaxation tests are given in Figures S9 and S10. Figure 5 shows representative stress–relaxation behaviors of PUEs. Table S1 summarizes the relaxation times at which the storage moduli reached 1/e of their initial values. It was confirmed that the relaxation time decreased as the concentration of azomethine groups in PUEs increased. AMD3-20 and AMD3-30 underwent stress relaxation earlier than AMD2-30 and AMD2-40, even though the azomethine group concentrations were comparable. Notably, the distribution and concentration of azomethine groups strongly affect stress relaxation in the AMD-based PUs. Figure 6 shows the Arrhenius plots of relaxation times for PUEs based on AMD [33,34]. The relaxation times of AMD-based PUEs indeed fit the Arrhenius law and the activation energy, which was calculated based on slope, decreased as the azomethine concentration of PUEs based on AMD increased or became denser, as given in Table S1.

### 2.3. Mechanical Properties of PUEs Based on AMD

**Figure 7:**
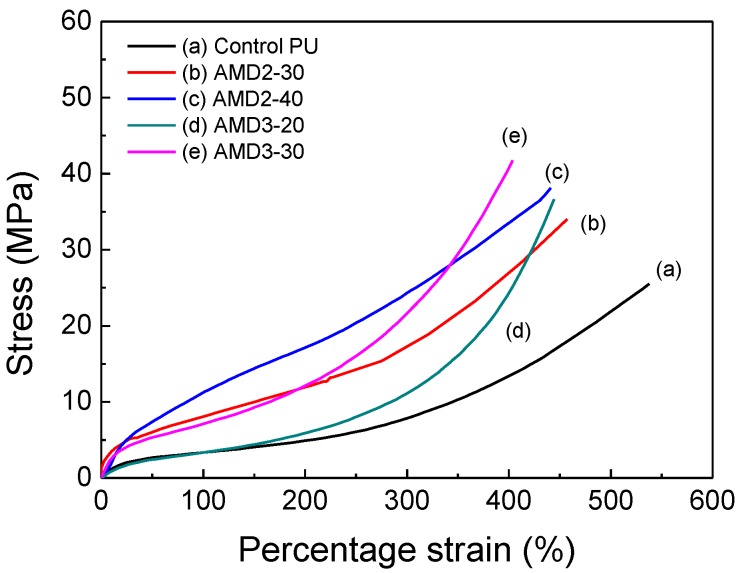
Typical stress–strain curves of PUEs based on AMDs.

**Figure 8:**
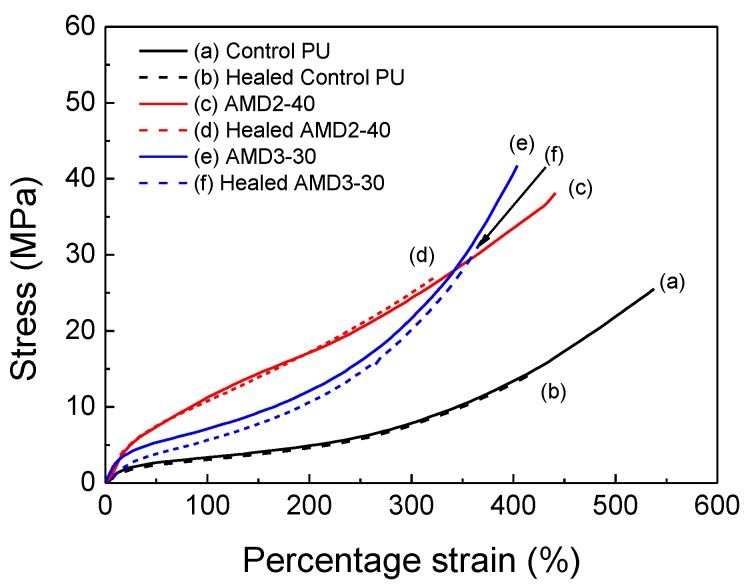
Stress–strain curves for AMD-based PUEs before (solid line) and after healing (broken line).

The stress–strain properties of PUEs were studied by tensile tests and are given in Figure S11. It is interesting to note that the tensile strengths and moduli of PUEs improved, while the elongation at break was lowered as the AMD content increased. Figure 7 shows representative tensile properties for two sets of PUEs with similar azomethine group concentrations. PUEs based on AMD3 showed more strain hardening than those based on AMD2. Although AMD-based PUs in this study showed earlier stress relaxation at elevated temperatures above 120 °C, the tensile properties of the systems at room temperature were superior to those of the control PUE. A dog bone specimen was prepared to measure the self-healing properties. The center of the prepared specimen was cut with a knife and the two halves were immediately reattached. The specimen was placed in a convection oven at 130 °C for 30 min. Scheme S1 was supplemented with a self-healing scheme by the metathesis of azomethine. The tensile properties of the healed specimen at elevated temperatures after cutting for PUEs based on AMDs were studied and are summarized in Table S2. The healing efficiency of PUEs increased with the concentration of azomethine groups in the PUEs. The healing efficiencies of AMD3-20 and AMD3-30 were higher than those of AMD2-30 and AMD2-40. Figure 8 shows the tensile properties of representative PUEs before and after healing. The healing of PUEs based on AMD3 is more effective than that of those based on AMD2. Figure S12 shows visual images of the samples before and after healing, confirming the self-healing of the specimens. Results of repetitive self-healing are given in Figure S13. Healing efficiencies of AMD3-30 showed gradual decrease from 76% to 56% in the three repeated healing tests. Further studies on the effects of solvents, different rates, and sample thickness on the self-healing of AMD based PUs are necessary for various applications in the future. Figure S14 illustrates the adhesion test between different specimens. AMD2-30 specimens were tested for adhesion with control PU, AMD3-20, and AMD3-30 specimens. As a result, adhesion test with AMD2-30 and AMD3-20 or AMD3-30 containing azomethine group showed higher tensile strength than that with AMD3-20 and control PU. Control PU also displayed 50% tensile strength of the initial control PU due to the healing by the diffusion of soft segment domains and hydrogen bonding. Adhesion tests with AMD-based PUEs showed 74–76% tensile strength of the initial AMD-based PUEs because the diffusion of the soft domain and hydrogen bonding and the azomethine metathesis proceeded from the surface of the specimens.

### 2.4. Rheological Properties of AMD-Based PUs

**Figure 9:**
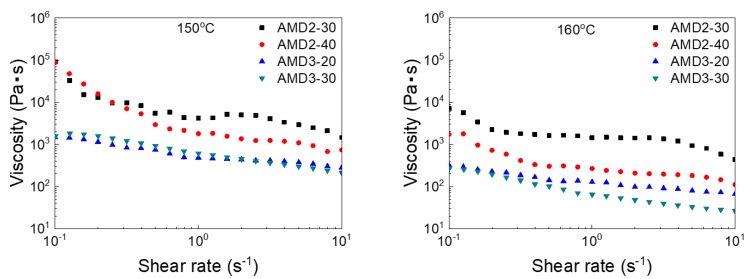
Flow curves of AMD-based PUEs at: 150 °C (**left**); and 160 °C (**right**).

Figure 9 shows flow curves of representative PUEs. The viscosities of PUEs decreased as the concentration of azomethine groups increased due to azomethine metathesis. The control PU could not be measured below 170 °C. Flow curves of PUEs are also presented in Figures S15 and S16 at various temperatures. The viscosities of PUEs based on AMD3 are lower than those of PUEs based on AMD2 even though their average molecular weights are comparable.

## 3. Materials and Methods

### 3.1. Materials

Terephthalic aldehyde (TA, MW: 134.13, 99%), ethanolamine (EA, MW: 61.08), poly (tetramethylene ether) glycol (PTMEG, MW: 1000, 99%), and 1,4-butanediol (BDO; 99%) were obtained from Sigma–Aldrich (Youngin, Korea). Commercially available polyether diamine (Jeffamine 230, MW: 230, 99%) was purchased from Huntsman, and 4,4′-methylene diphenyl diisocyanate (MDI, 99%) was obtained from BASF.

### 3.2. Preparation of AMDs

The synthesis of azomethine diols were progressed by condensation reaction between amine and aldehyde [24,25,26,27]. AMD was synthesized via condensation with TA (13.4 g, 0.1 mol), polyether diamine (11.5 g, 0.05 mol), and EA (12.2 g, 0.2 mol). The reaction progressed stepwise. First, 0.1 mol of TA was reacted with 0.05 mol of polyether diamine at 80 °C for 5 h in an evaporator at reduced pressure. Then, 0.2 mol of EA was added to the reactant. The condensation reaction between the reactant and EA was maintained at 80 °C for 5 h in the evaporator at reduced pressure, and the AMD obtained was designated as AMD2. AMD of different molecular design, AMD3, was also synthesized via condensation with TA (13.4 g, 0.3 mol), polyether diamine (11.5 g, 0.2 mol), and EA (12.2 g, 0.2 mol). The reaction progressed via three steps. In the first step, 0.1 mol of TA was reacted with 0.2 mol of polyether diamine. In the second step, 0.2 mol of TA was added to the first reactant. Finally, 0.2 mol of EA was added to the second reactant. Each step progressed at 80 °C for 5 h in an evaporator at reduced pressure. For all the AMD syntheses, the complete consumption of primary amine was confirmed via amine titration following ASTM D2074.

### 3.3. Preparation of Control PU and AMD-Based PUs

AMD-based PUs were synthesized via a prepolymer method. The prepolymer was synthesized by reacting 0.1 mol of PTMEG and 0.2 mol of MDI at 70 °C for 3 h. The synthesized prepolymer (20 g) was mixed with the desired amounts of MDI, BDO, and AMD. After degassing, the mixture was poured into a glass mold and cured at 110 °C for 24 h. Scheme S2 presents the structure of AMD based PUE.

### 3.4. Characterization

Fourier transform infrared (FT-IR) spectroscopy (FT-IR-302 from Jasco, Tokyo, Japan) was employed to confirm the synthesis of AMD and AMD-based PUs. ^1^H-NMR spectra were recorded in DMSO-*d*_6_ with a Bruker AM400 spectrometer (400 MHz). The molecular weight of AMD-based PUs was determined using GPC (Agilent 1200S from Agilent, Palo Alto, CA, USA) employing an RI detector (Optilab rEX from Wyatt, Santa Barbara, CA, USA). The samples were dissolved in DMF/THF (1:1 by wt.), and polystyrene standards was used for universal calibration. The flow rate for GPC measurement was 1 mL/min. The thermal properties of AMD-based PUs were measured using DSC (Q20 from TA Instruments, Seoul, Korea). The samples were initially cooled to −90 °C and maintained at this temperature for 1 min. The ramp rate for all the measurements was maintained at 10 °C/min up to 240 °C. A second scan was conducted under the same conditions. All the tests were performed in a N_2_ atmosphere. The dynamic mechanical properties and relaxation times of AMD-based PUs were measured by DMA, Q800 from TA Instruments) using a film tension clamp. Films were carefully cut into sections of 5.3 mm in width. The tests related to the dynamic mechanical properties were performed between −90 °C and 240 °C at a heating rate of 5 °C/min. Relaxation tests were also performed at 120 °C, 130 °C, 140 °C, and 150 °C for 20 min. For the relaxation tests, an axial force of 0.01 N and a deformation of 1% were applied. Liquid chromatography-mass spectrometry (LC-MS; AGILENT 1100, Agilent, Palo Alto, CA, USA) was performed to confirm the exchange reaction between the azomethine groups in the model compounds. The azomethine model compound based on vanillin and hydroxybenzylaldehyde were dissolved in ethanol at a molar ratio of 1:1; then, the two solutions were mixed and stirred at 60 °C for 5 h. The solvent was then completely removed in a vacuum oven to get model compounds that underwent azomethine metathesis. A universal testing machine (UTM; LR5K from Lloyd, Suwon-City, Korea) was used to measure the tensile strength and percentage elongation. The test followed the ASTM-D638 method. Rheological measurements were performed using a controlled-stress rheometer (AR2000 from TA Instruments) equipped with parallel plates of 25-mm diameter at various temperatures in N_2_ atmosphere. A strain sweep was performed at strains from 0.1% to 10% at a constant frequency of 1 rad/s to determine the linear viscoelastic region. SAXS (D8 DISCOVER, BRUKER, Seongnam-si, Korea) patterns were collected at room temperature using Cu-Kα radiation (λ = 1.541 Å), at a voltage of 50 kV and current of 1000 µA, over the Bragg angle (2θ) range of 0−9°. The phase separation of the synthesized PUEs was probed by atomic force microscopy (AFM; Digital Instruments, Nanoscope IV A, Goleta, CA, USA) in tapping mode.

## 4. Conclusions

In this study, we investigated the self-healing of AMD-based PUEs via the metathesis reactions of azomethine groups, which can be efficiently exploited for the self-healing of PUEs. Particularly, the influence of the azomethine group concentration and distribution on self-healing properties was investigated via the thermal and rheological properties. As the AMD content of polyols increased from 10% to 40%, a decrease in the relaxation time of PUE was observed in all specimens. The relaxation time of AMD2 based PUEs decreased significantly from 186 to 53 s at 140 °C. In AMD3 based PUEs, its relaxation time decreased from 129 to 73 s at 130 °C. Thus, the distance between the azomethine groups affected the self-healing properties. As the distance between the azomethine groups decreased, the metathesis reactions proceeded more easily. Therefore, the distribution of the azomethine groups as well as their concentration significantly affected the self-healing. AMD2 was designed to achieve a more even distribution of azomethine groups in AMD-based PUEs than AMD3. However, AMD3 was designed to achieve a dense distribution of azomethine groups in AMD-based PUEs. In conclusion, a high concentration of azomethine groups produces better flow properties and a shorter relaxation time. For AMD2-30 and AMD3-20, or AMD2-40 and AMD3-30, with similar concentrations of azomethine groups, the relaxation time of AMD3 systems, designed with a more intensive distribution, was shorter than that of AMD2 systems. Our experimental results show that the flow properties are also improved under conditions favoring the azomethine exchange reaction. AMD based PUEs are mechanically robust at room temperatures while they show self-healing properties and relatively low viscosities at elevated temperatures. Potential applications of AMD based PUEs are hot-melt adhesives and thermoplastic elastomers.

## Figures and Tables

**Table 1:** Sample code and composition of synthesized PUEs.

Sample Code	Composition (Molar Ratios)				Content of Azomethine (mmol/g)
	Azomethine Diol (mol)	PTMEG 1000 (mol)	MDI (mol)	1,4-BD (mol)	
Control PU	-	1	2	1	0
AMD2-10	0.1	0.9	2	1	0.26
AMD2-20	0.2	0.8	2	1	0.53
AMD2-30	0.3	0.7	2	1	0.81
AMD2-40	0.4	0.6	2	1	1.13
AMD3-10	0.1	0.9	2	1	0.37
AMD3-20	0.2	0.8	2	1	0.77
AMD3-30	0.3	0.7	2	1	1.16
AMD3-40	0.4	0.6	2	1	1.56

**Table 2:** Molecular weights of synthesized PUEs.

Sample Code	Molecular Weight (g/mol)			Ð
	Mn	MW	Mz	
Control PU	17,500	31,300	46,700	1.78
AMD2-10	16,200	34,600	61,900	2.14
AMD2-20	12,900	30,800	66,300	2.40
AMD2-30	13,500	30,100	58,100	2.23
AMD2-40	10,000	23,300	42,600	2.33
AMD3-10	13,800	25,000	39,500	1.80
AMD3-20	13,700	32,500	66,700	2.37
AMD3-30	13,400	26,700	46,000	1.99
AMD3-40	13,100	26,300	44,900	1.99

## Supplementary Materials

The following are available online: Table S1: Relaxation time and activation energy of synthesized PUEs, Table S2: Tensile strength and healing efficiency of synthesized PUEs at 130 °C for 30 min, Table S3: Thermal properties of synthesized PUEs, Scheme S1: Schematic representation of the azomethine metathesis in AMD based PUE, Scheme S2: Schematic syntheses AMD based PUEs, Figure S1: ^1^H-NMR spectrum of AMD2, Figure S2: ^1^H-NMR spectrum of AMD3, Figure S3: Flow chart for the synthesis of AMD based PUEs, Figure S4: TGA thermograms of AMD based PUEs: (**A**) AMD2 based PUEs; and (**B**) AMD3 based PUEs, Figure S5: Dynamic mechanical analysis of AMD based PUEs: (**A**) Storage moduli of AMD2 based PUEs; (**B**) Storage moduli of AMD3 based PUEs; (**C**) Tan Delta of AMD2 based PUEs; and (**D**) Tan Delta of AMD3 based PUEs, Figure S6: DSC thermograms of AMD based PUEs: (**A**) AMD2 based PUEs; and (**B**) AMD3 based PUEs, Figure S7: SAXS data (absolute intensity profiles) of the synthesized PUEs, Figure S8: AFM images of synthesized PUEs, Figure S9: Representative stress relaxation behaviors for the AMD2 based PUEs, Figure S10: Representative stress relaxation behaviors for the synthesized AMD3 based PUEs, Figure S11: Stress–strain curves of the synthesized PUEs before and after healing at 130 °C for 30 min: (**A**) AMD2 based PUEs; (**B**) AMD3 based PUEs; (**C**) Healed AMD2 based PUEs; (**D**) Healed AMD3 based PUEs; (**E**) Tensile stress of synthesized PUEs; and (**F**) Toughness of synthesized PUEs, Figure S12: Images of self-healing test for AMD2-20, Figure S13: Stress–strain curves of the AMD3-30 after repeated healing at 130 °C for 30 min, Figure S14: Stress–strain curves of the adhered specimens of different PUEs after healing at 130 °C for 30 min, Figure S15: Flow curves of AMD2 based PUEs at different temperatures, Figure S16: Flow curves of AMD3 based PUEs at different temperatures.
