# Supplementary material for: Design of Azomethine Diols for Efficient Self-Healing of Strong Polyurethane Elastomers
Source: Molecules. 2018 Nov 9;23(11):2928. doi: 10.3390/molecules23112928 (PMC6278415; doi:10.3390/molecules23112928)
Supplement: Supplementary file 1 [file molecules-23-02928-s001.pdf]

Supplymentary Materials

Design of azomethine diols for efficient self-healing and strong polyurethane elastomers

Dae-Woo Lee †, Han-Na Kim †, and Dai-Soo Lee *

Division of Semiconductor and Chemical Engineering, Chonbuk National University, Baekjedaero 567, Jeonju 54896, Republic of Korea; dwlee2310@hanmail.net (D.W.L.); hnk07@hanmail.net (H.N.K.)

† Contributed equally to this work

* Correspondence: daisoolee@jbnu.ac.kr; Tel.: +82-63-270-2310

Table S1. Relaxation time and activation energy of synthesized PUs

Sample code	Relaxation time (s)					Activation energy [1)] (kJ/mol)
	110℃	120℃	130℃	140c	150℃	
**Control PU**	-[2)]	-	241[3)]	539	98	247.61
**AMD2-10**	-	1017	517	186	⦿[4)]	140.85
**AMD2-20**	-	685	198	83	⦿	136.35
**AMD2-30**	-	507	169	74	⦿	131.59
**AMD2-40**	-	409	114	53	⦿	131.11
**AMD3-10**	282[5)]	232	129	⦿	⦿	77.28
**AMD3-20**	611	174	92	⦿	⦿	82.97
**AMD3-30**	246	165	79	⦿	⦿	66.37
**AMD3-40**	176	159	73	⦿	⦿	56.41

1) The activation energy was obtained from the slopes of Arrhenius plots based on the equation, $\tau = Ae^{-E_a/(RT)}$, where A is the pre-exponential factor, R is the universal gas constant, T is the absolute temperature, and τ is the relaxation time.

2) '-' represents that the relaxation modulus values did not reach 1/e of the initial value.

3) Relaxation time of PUE at 145 ℃

4) '⦿' represents that the relaxation modulus could not be measured due to the softening of specimens.

5) Relaxation time of AMD3-10 at 115 ℃

Table S2. Tensile strength and healing efficiency of synthesized PUEs at 130 °C for 30 min

**Sample code**	**Tensile strength (MPa)**		**Healing efficiency* (%)**
	**before healing**	**after healing**	
**Control PU**	26	15	57
**AMD2-10**	30	18	61
**AMD2-20**	32	21	66
**AMD2-30**	34	24	70
**AMD2-40**	38	27	72
**AMD3-10**	23	16	71
**AMD3-20**	37	27	73
**AMD3-30**	42	32	77
**AMD3-40**	51	39	78

* Healing-efficiency was defined by the equation, $\sigma_{healed}/\sigma_{pristine} \times 100$, where $\sigma_{pristine}$ is the tensile strength of pristine specimen and σ_{healed} is the tensile strength of specimen after the healing.

Table S3. Thermal properties of synthesized PUEs

**Sample code**	T_{gs} (℃) 1)	5% T_d (℃) 2)	10% T_d (℃) 2)	T_{flow} (℃) 3)
**Control PU**	-51	323	343	152
**AMD2-10**	-24	308	326	148
**AMD2-20**	-7	295	319	148
**AMD2-30**	3	280	309	145
**AMD2-40**	6	267	294	124
**AMD3-10**	-23	306	328	142
**AMD3-20**	3	290	313	136
**AMD3-30**	9	269	298	131
**AMD3-40**	18	254	283	122

1) Values measured by DSC

2) Values measured by TGA

3) Values measured by DMA

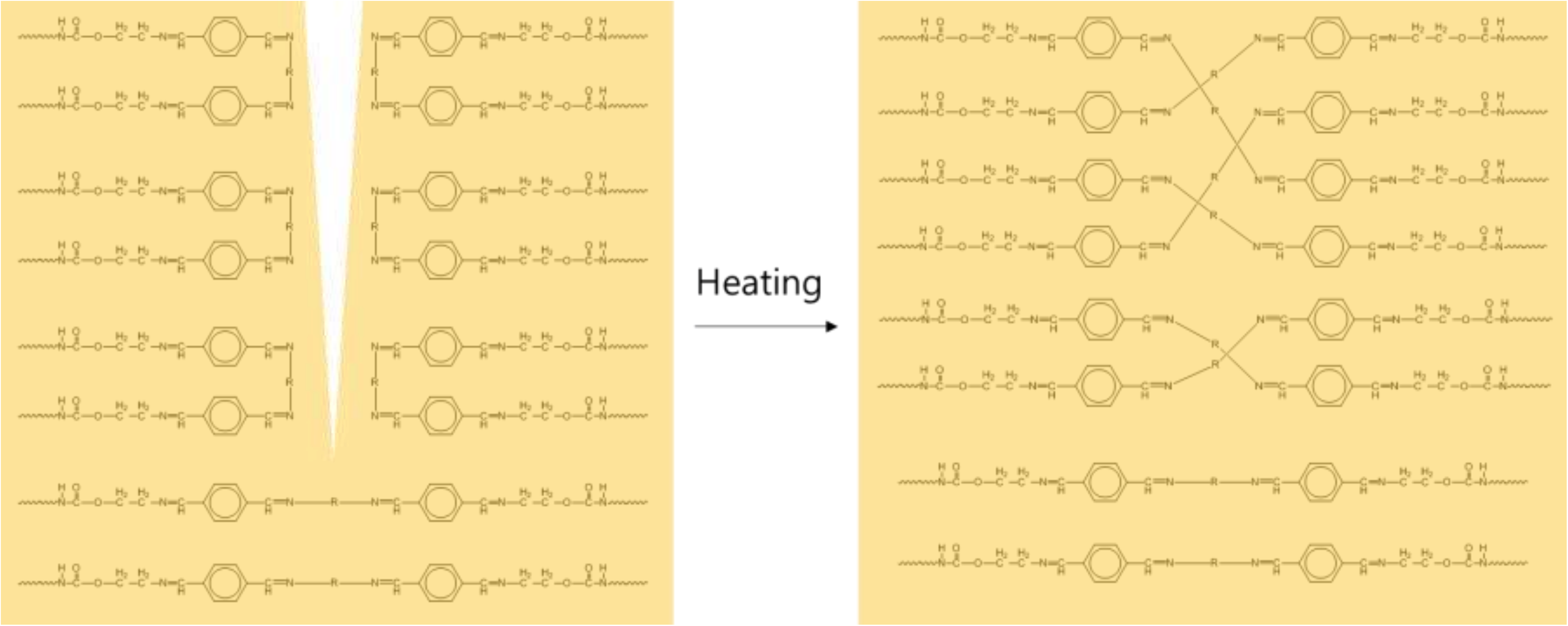

Scheme S1. Schematic representation of the azomethine metathesis in AMD based PUEs.

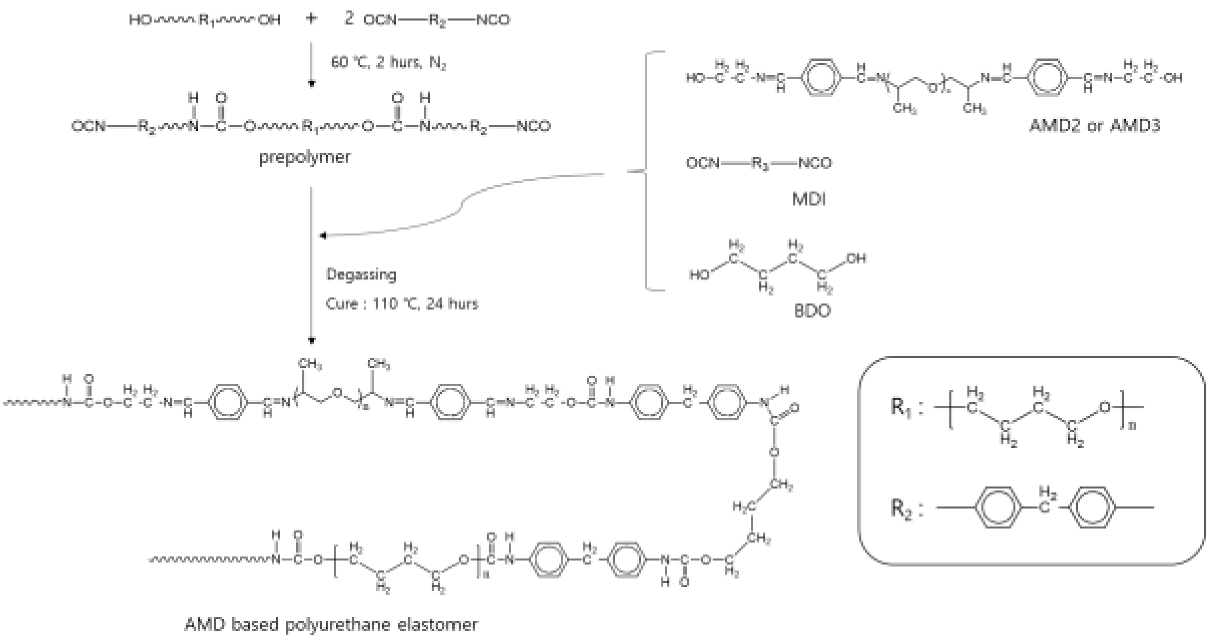

Scheme S2. Schematic syntheses of AMD based PUEs.

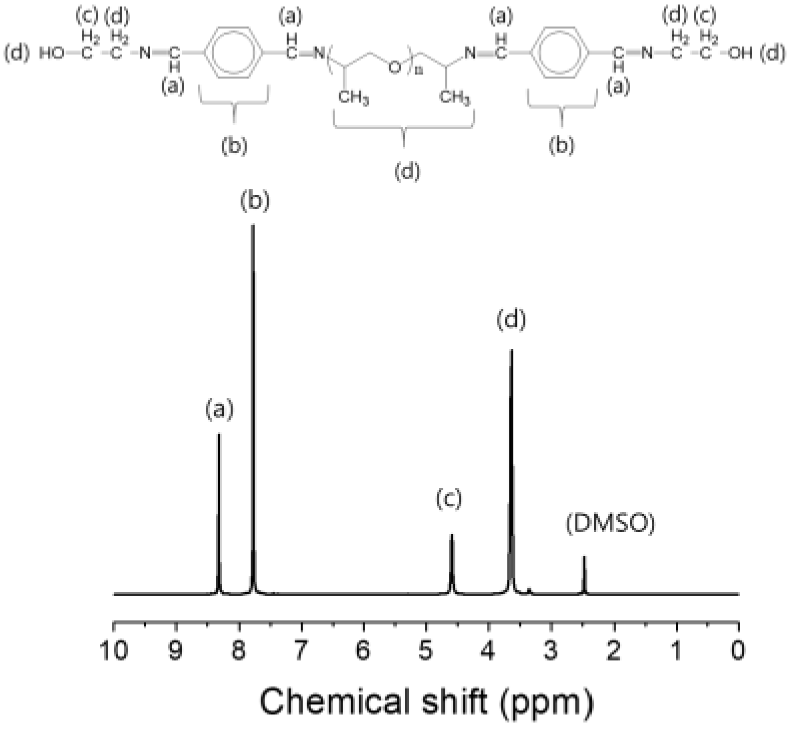

Figure S1. ^{1}H-NMR spectrum of AMD2.

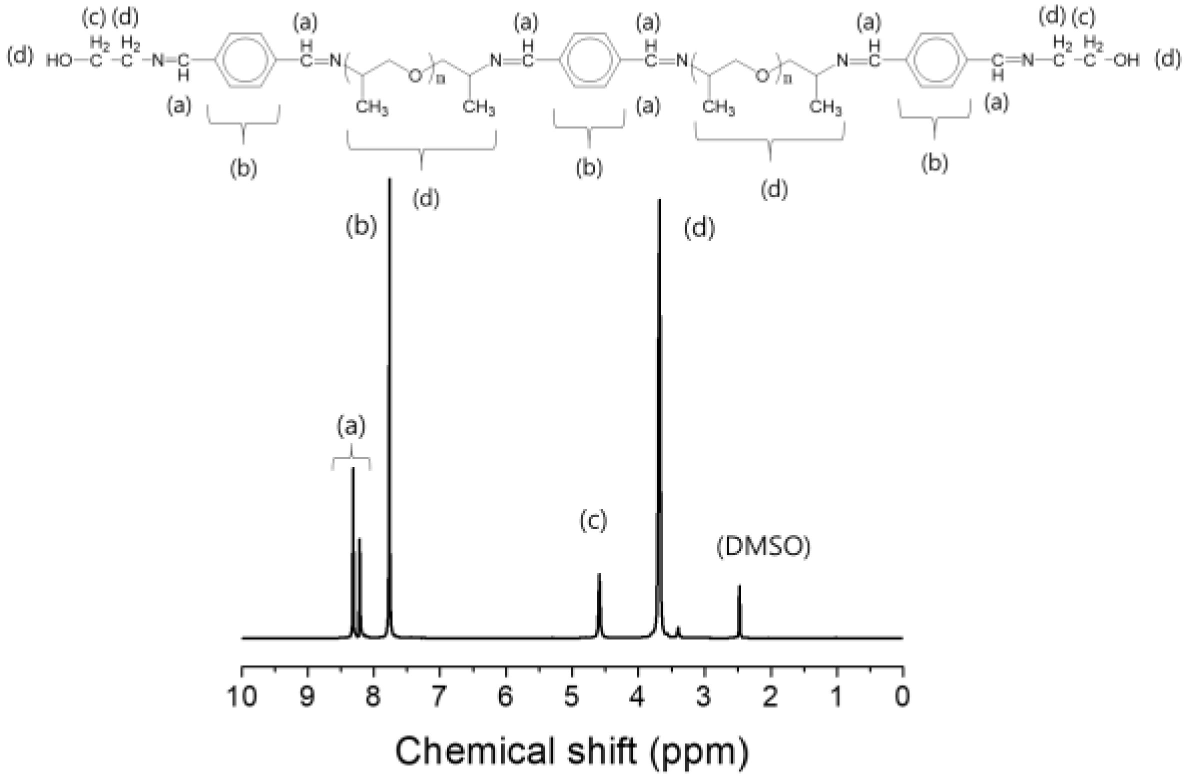

Figure S2. . ^{1}H-NMR spectrum of AMD3.

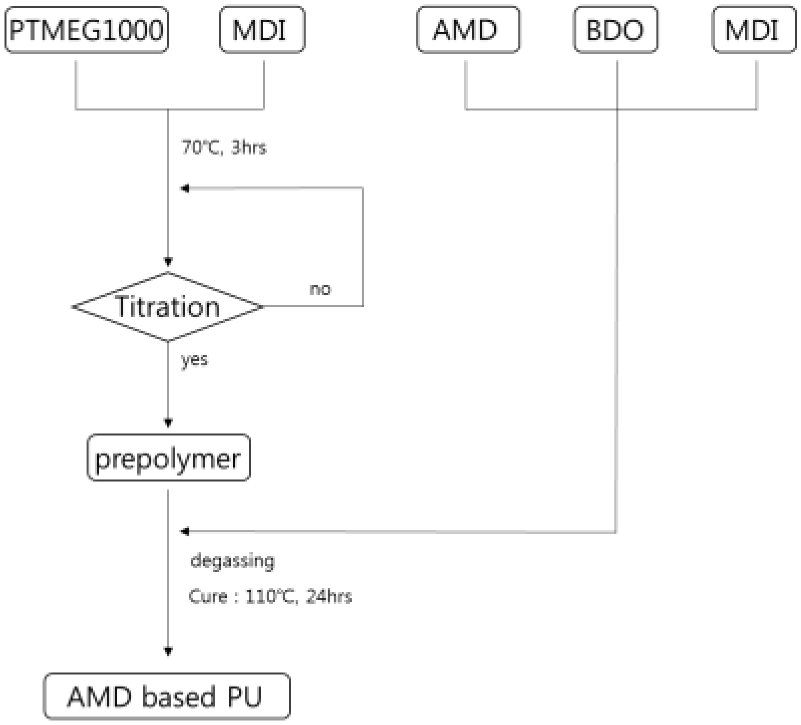

Figure S3. Flow chart for the synthesis of AMD based PUEs.

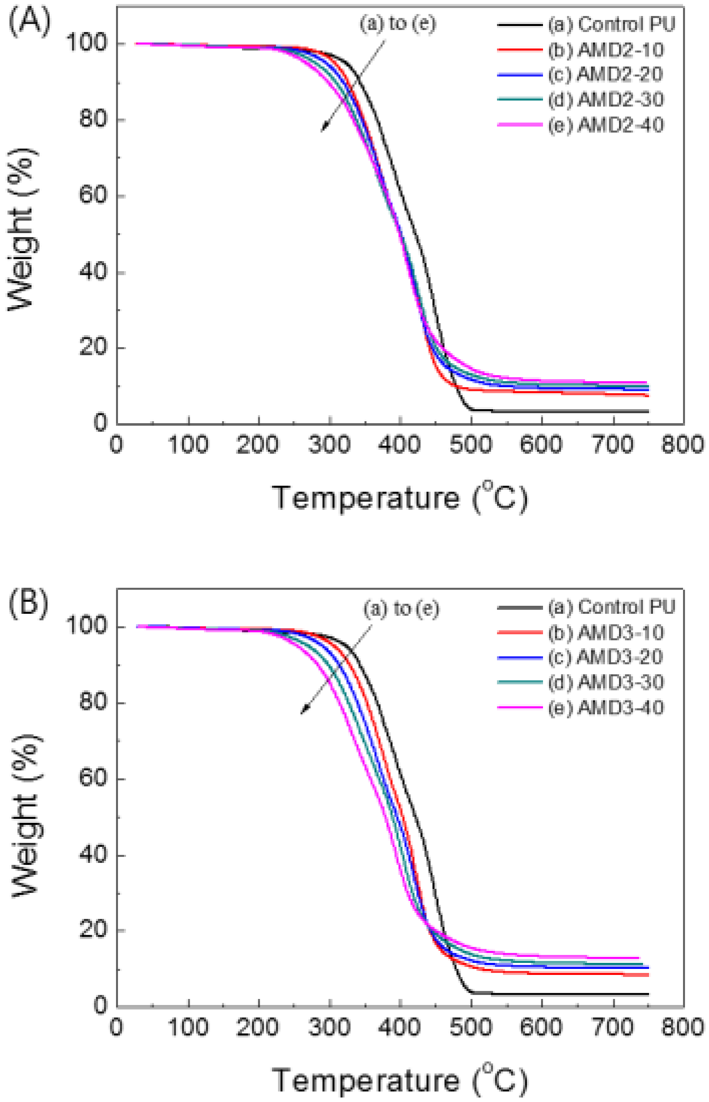

Figure S4. TGA thermograms of AMD based PUEs: (A) AMD2 based PUEs; (B) AMD3 based PUEs.

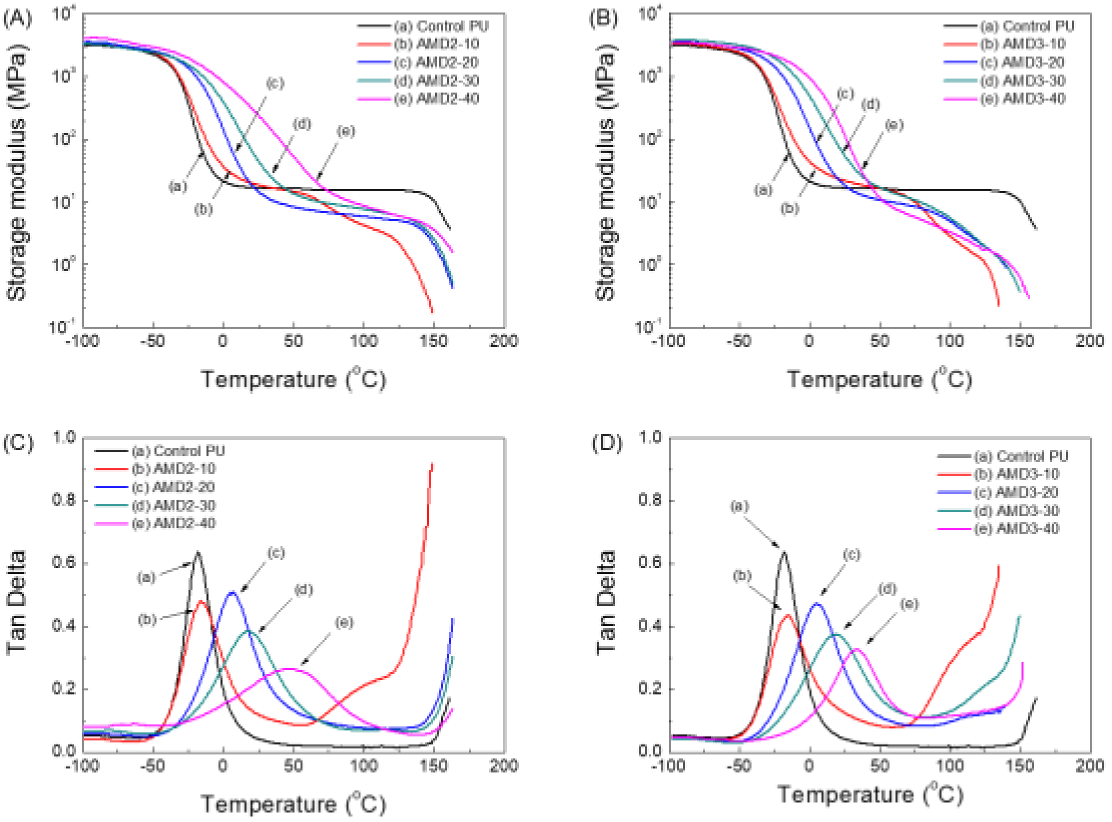

Figure S5. Dynamic mechanical analysis of AMD based PUEs: (A) Storage moduli of AMD2 based PUEs; (B) Storage moduli of AMD3 based PUEs; (C) Tan Delta of AMD2 based PUEs, (D) Tan Delta of AMD3 based PUEs.

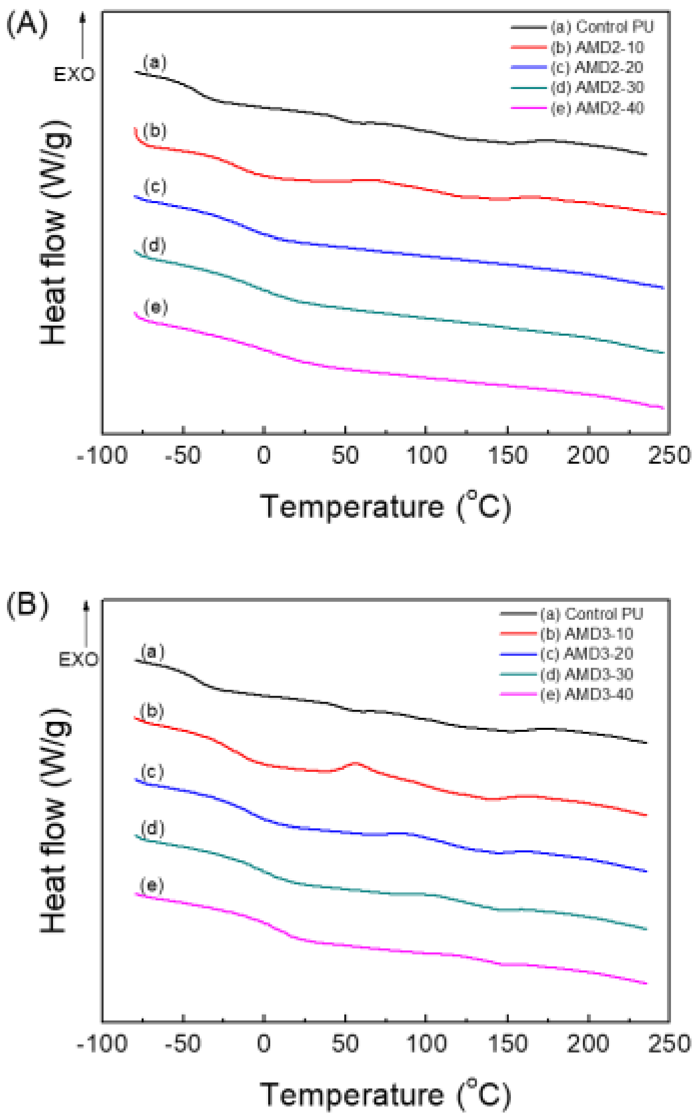

Figure S6. DSC thermograms of AMD based PUEs: (A) AMD2 based PUEs; (B) AMD3 based PUEs..

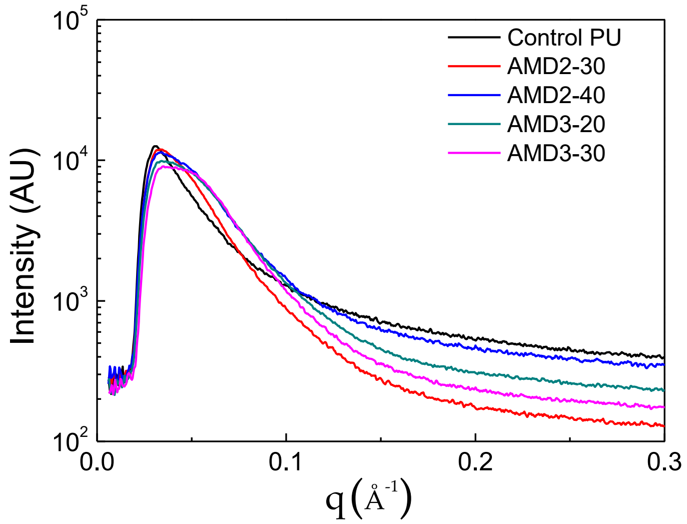

Figure S7. SAXS data (absolute intensity profiles) of the synthesized PUEs.

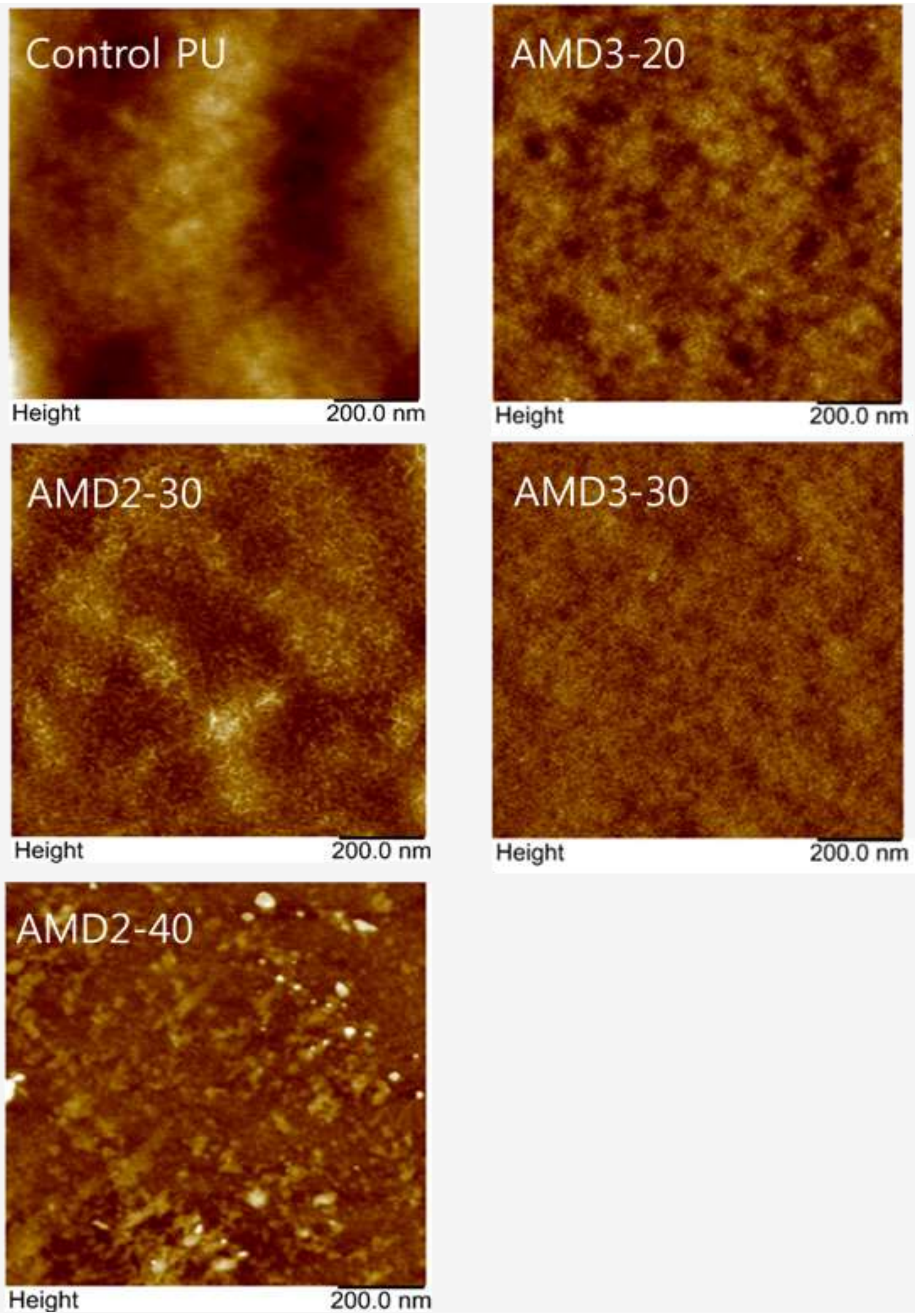

Figure S8. AFM images of synthesized PUEs.

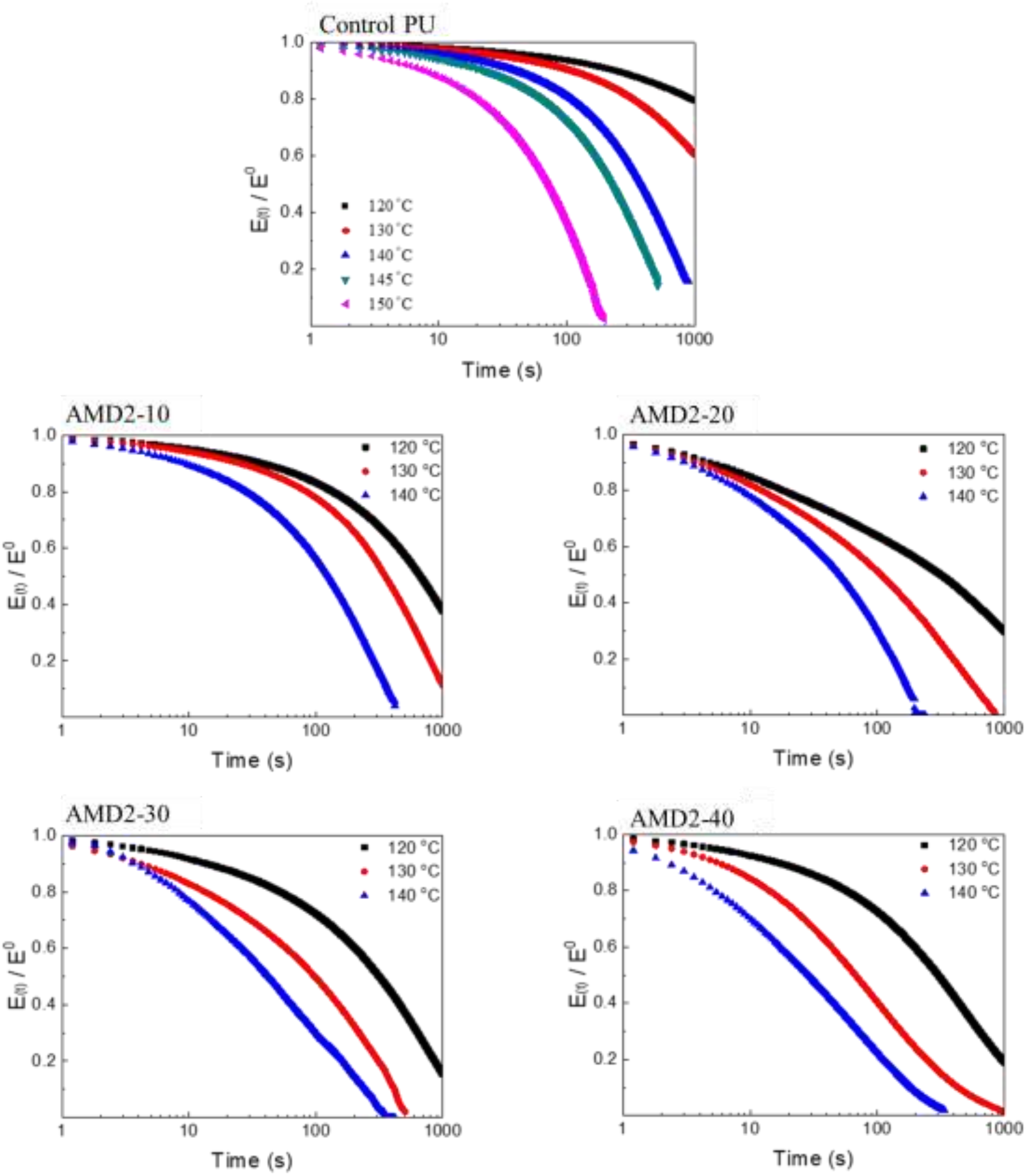

Figure S9. Representative stress relaxation behaviors of AMD2 based PUEs.

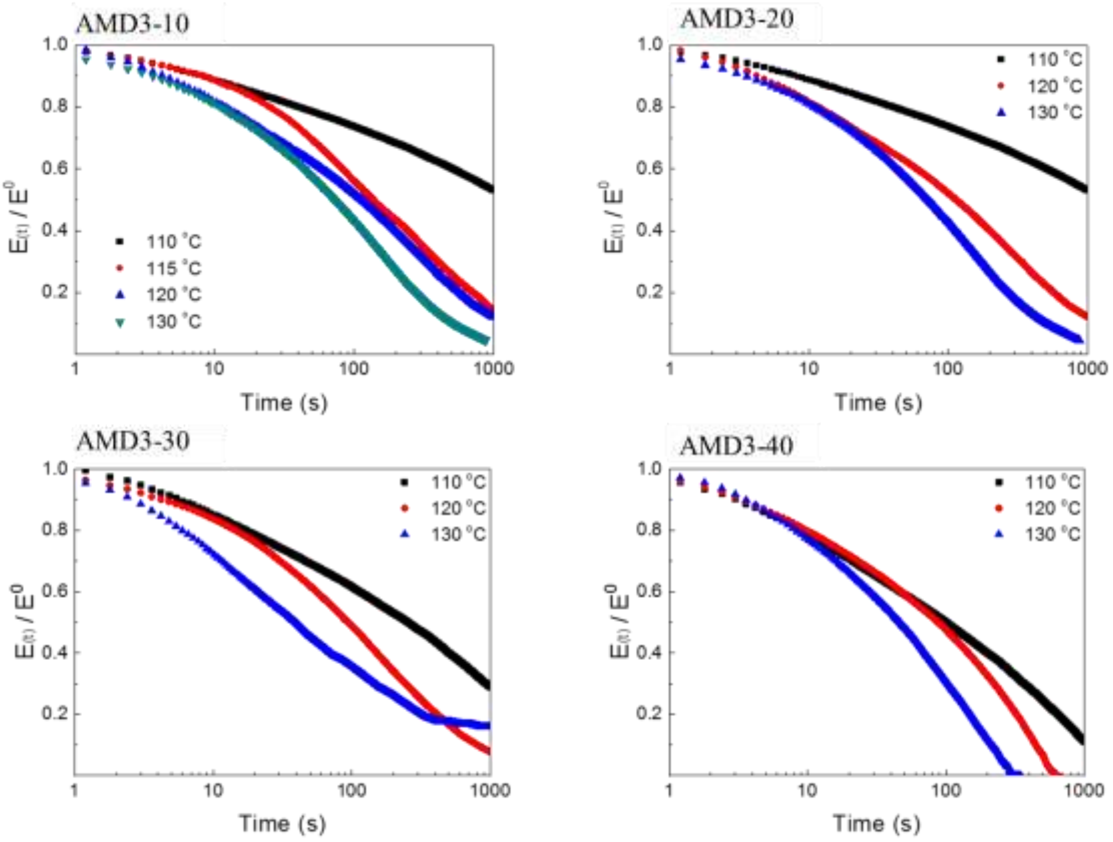

Figure S10. Representative stress relaxation behaviors of the synthesized AMD3 based PUEs.

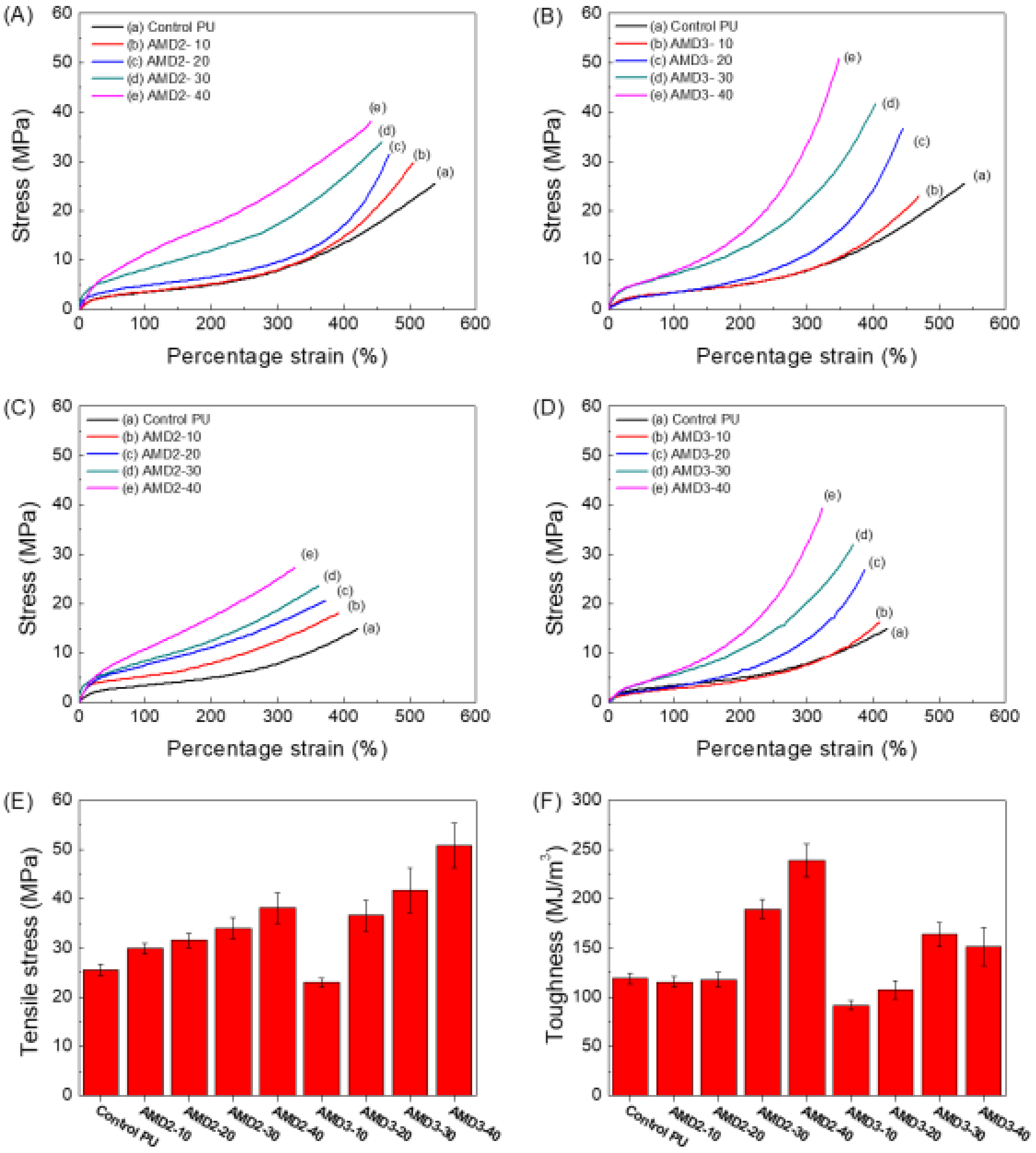

Figure S11. Stress strain curves of the synthesized PUEs before and after healing PUEs at 130 ℃ for 30 min: (A) AMD2 based PUEs; (B) AMD3 based PUEs; (C) Healed AMD2 base PUEs; (D) Healed AMD3 based PUEs; (E) Tensile stress of synthesized PUEs; (F) Toughness of synthesized PUEs.

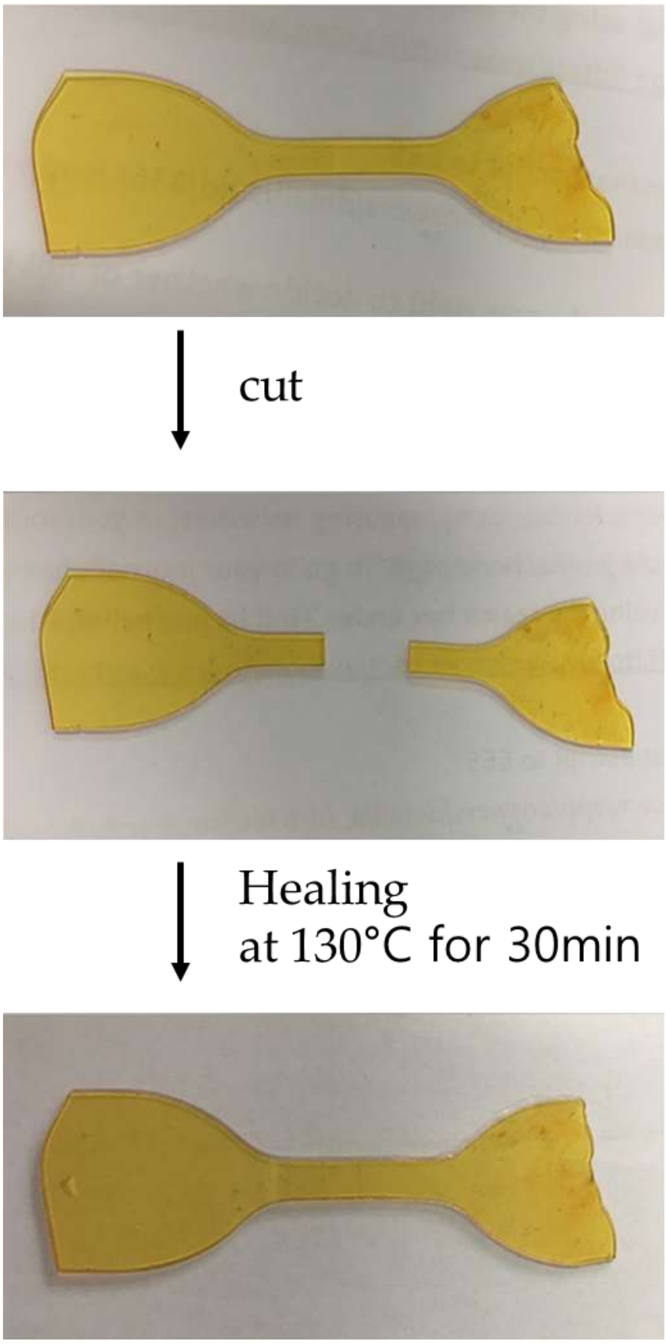

Figure S12. Images of self-healing test for AMD2-20.

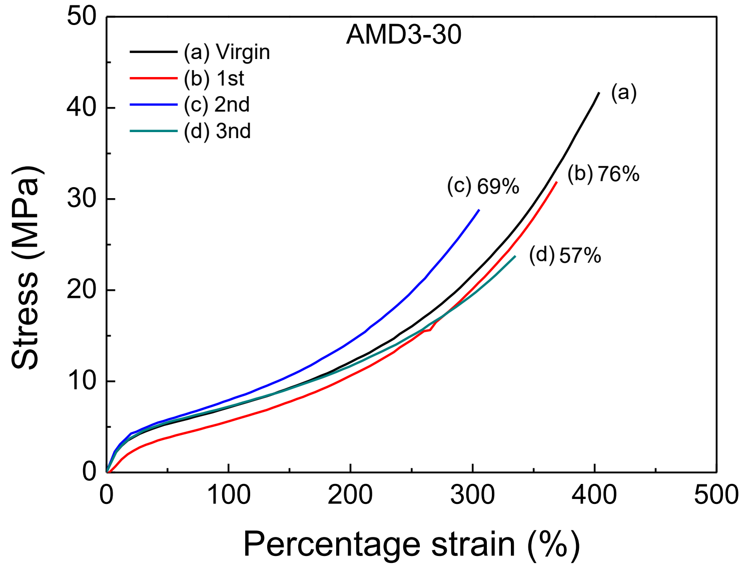

Figure S13. Stress-strain curves of the AMD3-30 after repeated healing tests at 130 ℃ for 30 min.

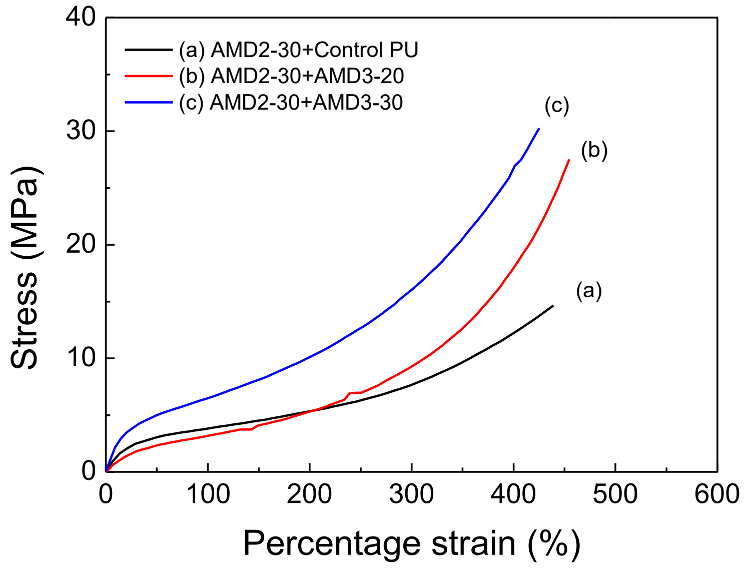

Figure S14. Stress-strain curves of the healed specimens of different PUEs after healing at 130 ℃ for 30 min.

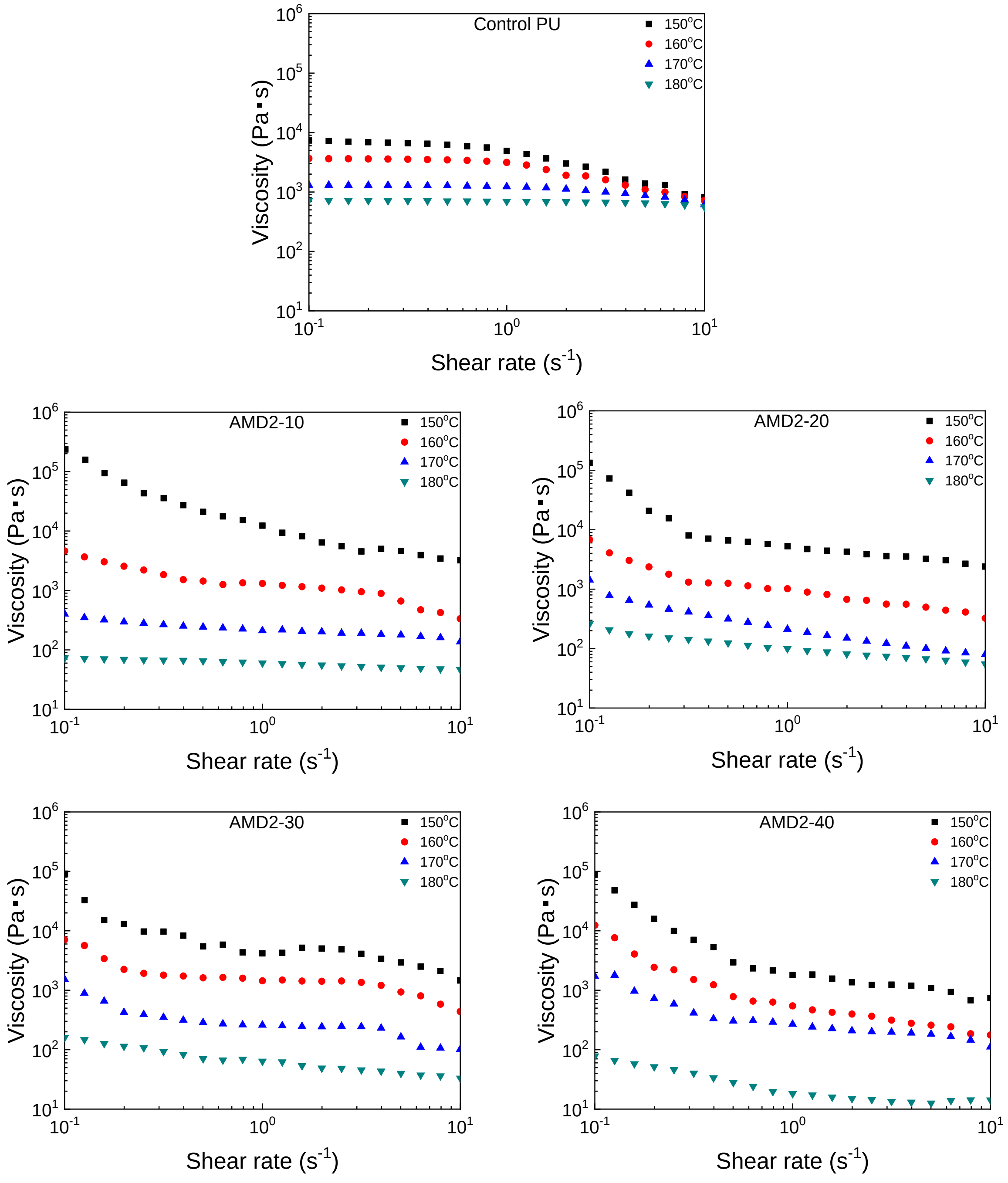

Figure S15. Flow curves of control PU and AMD2 based PUEs at different temperatures.

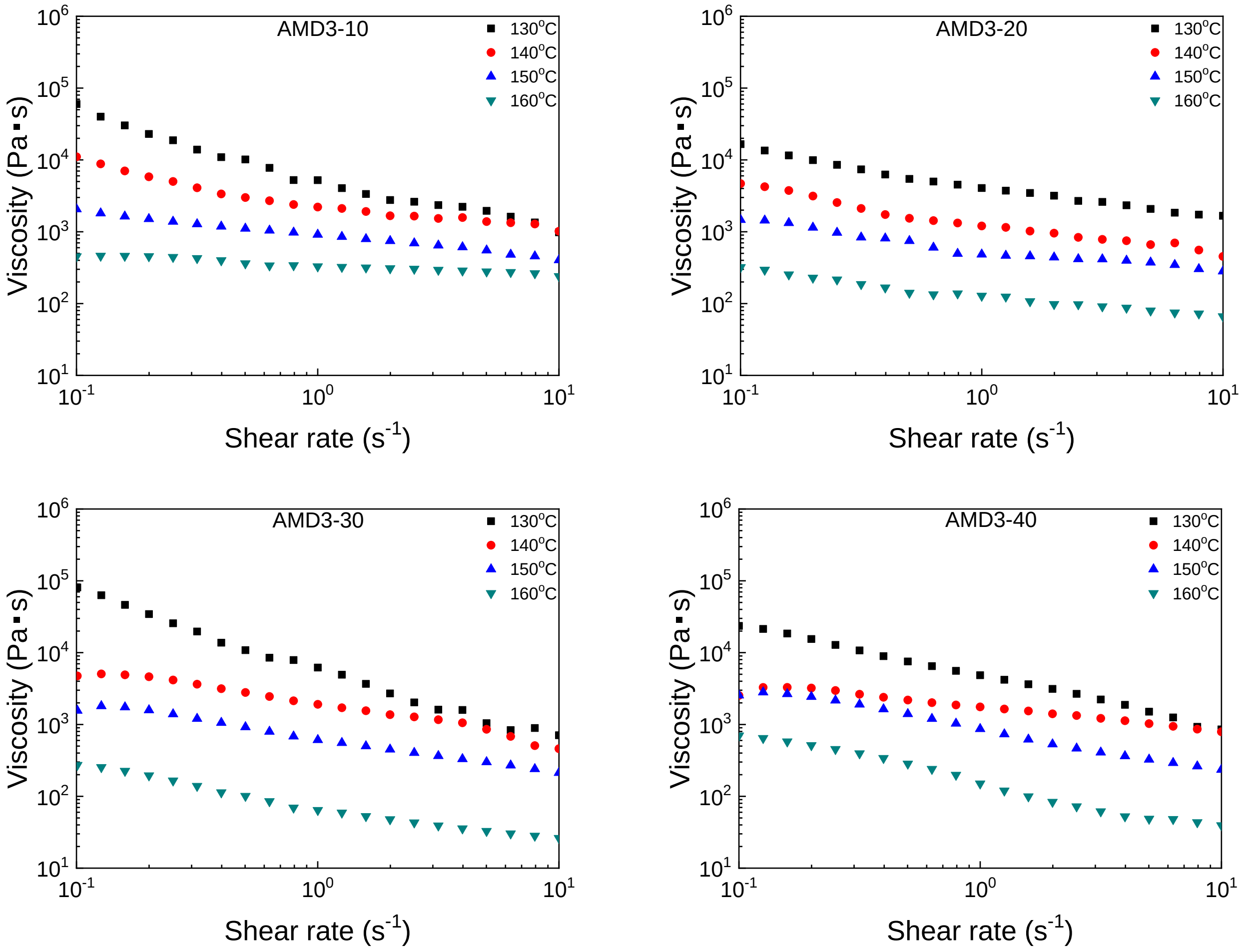

Figure S16. Flow curves of AMD3 based PUEs at different temperatures.
